# Interferometric-Based Vital-Sign Signature Identification with ML Validation for Privacy-Preserving Human Detection

**DOI:** 10.3390/s26185724

**Published:** 2026-09-09

**Authors:** Soumalya Bose, Jochen Bauer, Tobias Steigleder, Stefan G. Grießhammer, Julia Yip, Christoph Ostgathe, Jörg Franke, Georg Fischer

**Affiliations:** 1 Chair of Electronic Systems and Sensors (ESS), Institute of Electrical Engineering and Information Science, Brandenburg University of Technology Cottbus–Senftenberg, 03046 Cottbus, Germany; 2 Institute of Factory Automation and Production Systems, Friedrich-Alexander-Universität Erlangen–Nürnberg (FAU), 91058 Erlangen, Germany; 3 Department of Palliative Medicine, University Hospital Erlangen, Friedrich-Alexander-Universität Erlangen–Nürnberg (FAU), 91054 Erlangen, Germany; 4 Institute of Intelligent Technical Electronics and Systems, Friedrich-Alexander-Universität Erlangen–Nürnberg (FAU), 91058 Erlangen, Germany

**Keywords:** machine learning, intelligent system, interferometric radar sensing, privacy-preserving human detection, vital-sign signatures, smart cities, smart homes

## Abstract

Human presence detection is critical when building smart cities with use cases in sectors like smart homes, emergency evacuation, health-care monitoring and others. Existing human detection systems predominantly rely on camera-based imaging, raising privacy concerns. Moreover, conventional FMCW radar approaches are primarily motion-based, thus often failing to detect the presence of unconscious individuals, as in the case of search and rescue (SAR) operations. Some radar approaches use Doppler or spectral peak analysis to estimate respiration but fail to exploit phase coherence to resolve sub-millimeter chest displacement and higher-order physiological harmonics. This paper presents an interferometric radar framework that models multi-feature vital-sign signatures for human detection under controlled clinical settings using respiratory harmonic relationships, inter-harmonic consistency, chest-displacement spectral characteristics, and radar-derived cardiac mechanical signatures. Physiological relationships are used to establish the expected structure of the extracted features, while subject-to-subject variability and measurement uncertainty are used to determine practical acceptance regions from the training cohort. Experimental data from 30 healthy subjects were analyzed using a single interferometric radar sensor under controlled clinical conditions. The resulting signatures were subsequently evaluated using a machine-learning validation pipeline. With 243 test cases, the proposed framework achieved 89.71% accuracy, 95.26% precision, 94.15% F1-score, and 93.06% sensitivity. The study demonstrates that interferometric chest-displacement sensing can provide a privacy-preserving physiological feature space for human presence detection, while also identifying the limitations associated with unresolved multi-person signal superposition and hardware-induced phase uncertainty. Moreover, interferometric sensing by principle will work better than conventional radar approaches for SAR operations. Although validated in a controlled clinical environment, the framework establishes a foundational pathway towards future research for eventual deployment in next-generation smart systems.

## 1. Introduction

In 2023, Bloomberg cited research conducted by Grand View Research Inc., which projected the smart-home market to be approximately USD 445 billion by 2030 [1]. Advances in smart homes have accelerated research in the sub-domain of human detection, healthcare, and other areas. Major works from the past five years are shown in Table 1 and Table 2 that deal with human detection and healthcare monitoring using different modalities and applied in different used cases. Table 1 and Table 2 are organized chronologically and categorize studies by respective modality.

A significant portion of these works [2,3,4,5,6,7,8,9,10,11,12,13,14,15,16,17,18,19,20,21,22,23,24,25] deals with the use of cameras or multiple sensor modalities [31,32,33]. Although deep learning has improved accuracy detection, camera-centric approaches introduce privacy concerns and lack direct access to physiological information. Parallel efforts [26,27,28,29,30,31,32,33,34,35,36,37,38,39,40,41,60,61,62] have been made with the use of thermal cameras, infrared cameras, WiFi channel state information, LTE-4G, LiDAR, and RF. In these studies, the authors have successfully managed to detect humans while maintaining privacy. However, there is a need for separate sensors for healthcare monitoring, leading to increased inefficiency, computational complexity, cost, and energy usage of smart homes.

**Figure 1:**
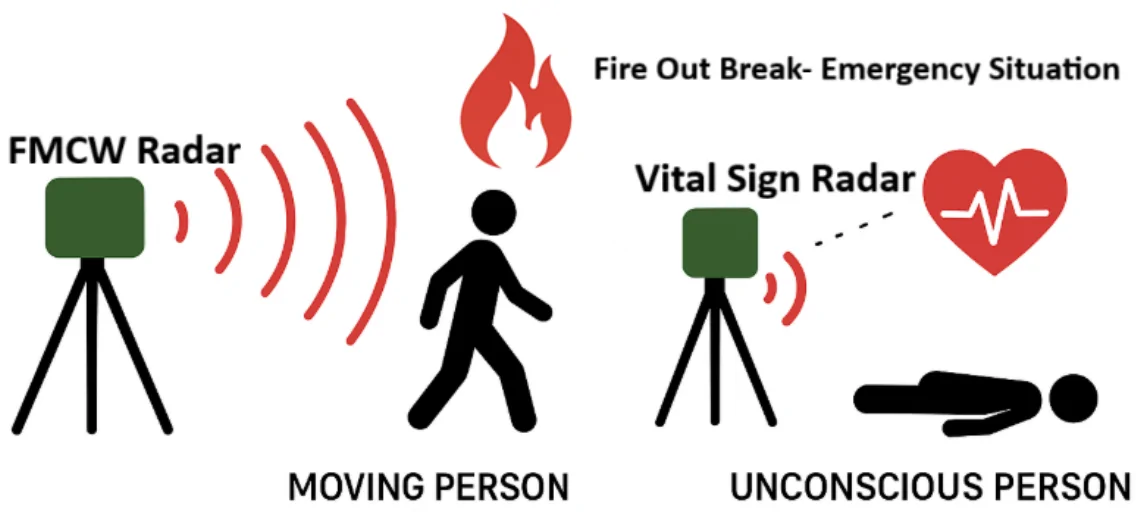
Advantage of interferometric vital-sign-based human detection over FMCW radar.

Over the past years, radar has been explored for detecting human motion using non-vital-sign methodologies which exhibit promising performance for moving subjects [42,43,48,51,52,53,54,56,57,58,59,59] but their reliability is questionable in detecting unconscious humans, such as in the case of emergency situations or search and rescue operation inside smart homes, as shown in Figure 1.

Going beyond presence detection, several recent works have investigated radar-based vital-sign monitoring [44,45,46,47,49,50,55], focusing on respiratory and heart rate information extraction using FMCW, SFCW, and UWB platforms. These research works solve efficiency, computational complexity, cost, and energy issues in smart homes by simultaneously detecting human subjects while maintaining privacy, with applications in healthcare monitoring and potential for use in SAR operations. However, these studies largely rely on single physiological features. Such single-signature frameworks remain vulnerable to physiological variability across subjects and contexts, including with respect to shallow breathing, temporary apnea, sensor misalignment, or acoustic masking of heart sounds. Human physiology is inherently dynamic and influenced by stress, activity level, and environmental conditions [63]. Consequently, reliance on a single vital sign does not provide the most robust human detection healthcare monitoring system in intelligent systems or for smart homes.

Table 1 and Table 2 highlight that there is a lack of unified frameworks that use multi-feature vital-sign signatures for privacy-preserving human detection and healthcare monitoring with the potential for use of such sensors in SAR operations.

Unlike conventional radar-based vital-sign monitoring that mainly uses range–Doppler or spectral peak analysis to infer breathing rate, interferometric sensing leverages phase coherence to detect sub-millimeter chest displacements and higher-order physiological harmonics. This capability enables the extraction of detailed cardio-respiratory signatures, including displacement amplitudes and inter-harmonic spacing, enabling robust human detection even under minimal motion or conditions of unconsciousness—scenarios where standard Doppler radar methods often fail. The authors of this work propose a multi-signature interferometric sensing framework that jointly exploits respiratory harmonics, inter-harmonic spacing, chest displacement, and heart sounds for reliable human detection under physiological variability. The authors implement a vital-sign signature-based machine-learning validation pipeline for a privacy-aware, energy-efficient human detection and healthcare system suitable for smart homes and intelligent systems.

The paper is organized as follows: Section 2 details the materials used in the study and elaborates on the research methodology. Section 3 provides the experimental results. Section 4 provides a discussion of the results. Last but not least, Section 5 concludes the research by considering the challenges faced by those conducting research in this area, the limitations of the study, along with discussion of the scope for future research.

## 2. Materials and Methods

The signal processing and feature extraction procedures are implemented using MATLAB (v2023a; MathWorks Inc., Natick, MA, USA), licensed by the University of Erlangen–Nuremberg (FAU), Germany.

### 2.1. Interferometric Sensing

Recent studies have demonstrated multi-person human detection using radar systems through clutter removal, angular separation, target tracking, and spatial filtering techniques, followed by respiration feature extraction [55]. However, the dataset used in [55] is not considered in this study. The characterization of physiologically consistent signatures requires artifact-free baseline measurements, which are difficult to obtain in spatially cluttered multi-person environments because of multi-path interference and signal overlap. The primary objective of this work is to identify robust multi-feature vital signs as signatures to detect human subjects so that a foundation can be laid for future work seeking to implement the proposed approach in real-world applications. Therefore, this study uses a less-complicated controlled single-subject dataset than the interferometric vital-sign dataset reported in [64]. In the dataset employed, vital signs were recorded in a clinically controlled environment. The controlled acquisition of data minimizes multi-path effects and enables reliable physiological signature extraction.

#### 2.1.1. Working Principle

The interferometric sensing system described in [64] is based on a passive six-port interferometric architecture operating as a continuous-wave (CW) radar at 24 GHz in the ISM band. Small movements along the radar line-of-sight produce phase variations between the transmitted (TX) and received (RX) signals. The displacement variation Δx(t) is related to the phase change Δϕ(t) as

(1) $$\Delta x\left(t\right)=\frac{\lambda }{4\pi }\Delta \phi \left(t\right),$$

where λ represents the wavelength of the transmitted electromagnetic wave.

The radar front-end generates in-phase (*I*) and quadrature (*Q*) baseband signals, which are simultaneously digitized using a high-resolution analog-to-digital converter (ADC). Hardware imperfections are compensated using ellipse reconstruction, after which, the instantaneous phase is calculated through arc-tangent demodulation as shown below:

(2) $$\phi \left(t\right)=\tan^{-1}\left(\frac{Q(t)}{I(t)}\right).$$

In an ideal quadrature receiver, the baseband signal can be represented as

(3) I(t)=A(t)cosϕ(t),Q(t)=A(t)sinϕ(t).

Real radar front-ends deviate from this ideal model because of DC offsets, unequal I/Q gain, quadrature phase error, amplitude fluctuations, ADC quantization, thermal noise, and residual phase noise. A more general representation is

(4) $$\left[\begin{array}{c} I_{m}\left(t\right)\\ Q_{m}\left(t\right) \end{array}\right]=H\left[\begin{array}{c} A(t)\cos\phi (t)\\ A(t)\sin\phi (t) \end{array}\right]+b+n\left(t\right),$$

where H represents gain imbalance and quadrature distortion, b represents DC offsets, and n(t) represents additive measurement noise. These non-idealistic components distort the ideal circular I/Q trajectory into an offset ellipse.

The ellipse-reconstruction procedure described in the dataset pre-processing compensates the dominant deterministic geometric distortion before phase demodulation. Nevertheless, residual calibration error and stochastic noise remain and propagate into the estimated phase. For small phase perturbations, the displacement error is approximately

(5) $$\delta x\left(t\right)=\frac{\lambda }{4\pi }\delta \phi \left(t\right).$$

Thus, the phase error is linearly transferred into displacement error through the interferometric conversion factor λ/(4π). The conversion in Equation (1) should therefore be interpreted as the ideal physical mapping, while the measured displacement includes residual hardware and demodulation uncertainty after calibration.

Phase unwrapping is subsequently applied to obtain continuous displacement tracking. The resulting displacement signal contains low-frequency respiratory components and higher-frequency cardiac micro-vibrations, thereby enabling contactless vital-sign monitoring. The digitized samples are buffered using an embedded micro-controller and transferred to a host computer for storage and further analysis. A synchronization sequence generated by the micro-controller is recorded simultaneously to ensure temporal alignment with external reference signals.

#### 2.1.2. Dataset

The dataset introduced in [64] consists of measurements of 30 healthy participants, including 16 female and 14 male subjects. The average age is 30.7±9.9 years, with a mean body mass index (BMI) of 23.2±3.3 kg/m^2^. During acquisition, chest displacement caused by respiratory and cardiac activity is recorded as phase variation between the transmitted and received radar signals. These phase measurements are subsequently converted into displacement signals using the known wavelength of the carrier frequency. Measurements are collected under clinically relevant conditions including Resting, Apnea, Valsalva, and Tilt-Up and Tilt-Down positions. This study considers only the Resting, Apnea, and Valsalva conditions because these measurements demonstrated higher interferometric signal quality compared with the tilted positions.

It is important to distinguish the number of subjects represented in the dataset from the number of simultaneously present subjects during an acquisition. The dataset contains 30 individual participants; however, each acquisition contains one participant at a time. Thus, the study evaluates inter-subject variability across 30 participants while intentionally excluding simultaneous multi-person radar returns. For two or more simultaneously illuminated subjects, the received interferometric signal can be approximated as a superposition of subject-dependent displacement components:

(6) $$x_{\mathrm{multi}}\left(t\right)=\sum_{m=1}^{M}\alpha_{m}x_{m}\left(t\right)+n\left(t\right),$$

where *M* is the number of subjects, $\alpha_{m}$ represents the propagation and radar-reflectivity-dependent contribution of subject *m*, and n(t) represents measurement noise and residual interference. The corresponding spectrum is therefore not generally equal to the spectrum of any individual subject. Overlapping respiratory frequencies can generate ambiguous or merged spectral peaks, while differences in amplitude and phase can further distort harmonic-feature estimation. Consequently, the thresholds derived in the present study should be interpreted as single-subject physiological signatures and not as a demonstrated multi-person separation mechanism. Multi-person operation requires an additional spatial, angular, range, or localization stage before individual vital-sign signatures can be reliably attributed to separate subjects.

### 2.2. Block Diagram of the Vital-Signs Signature-Based Novel Human Presence Detection System

**Figure 2:**
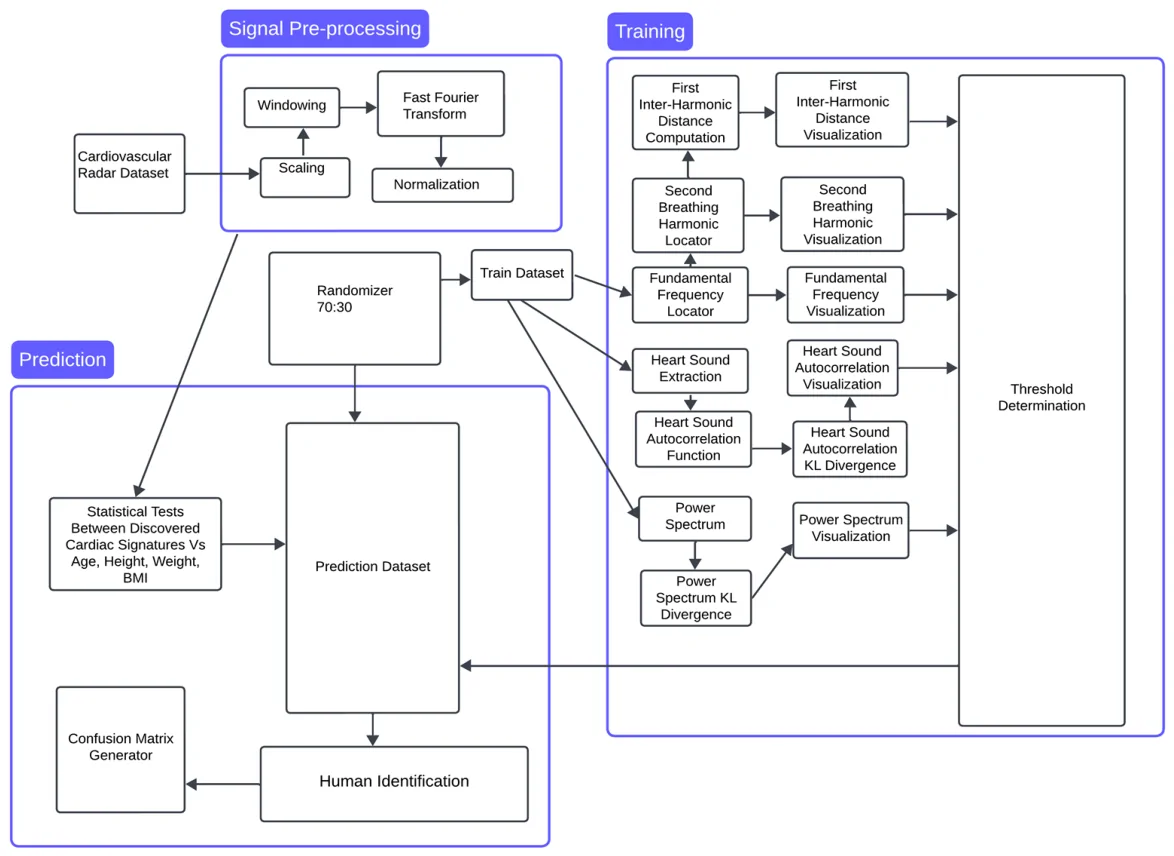
Vital-signs signature-based novel human presence detection system.

Figure 2 depicts the complete block diagram of the vital-sign signature identification with ML validation for human detection which will be discussed in the next sub-sections in detail.

### 2.3. Signal Pre-Processing

**Figure 3:**
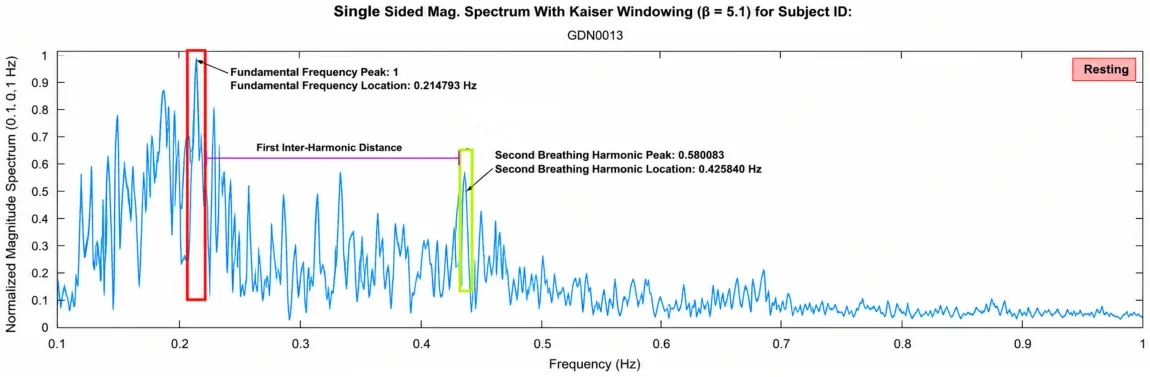
Processed interferometer signal for Subject ID: GDN0013 with vital-sign signatures.

In this section, the processes by which the interferometer signal is extracted from subjects in three clinically considered positions, for eventual removal of any remaining noisy artifacts, is discussed. An overview of this is shown in Figure 2. The interferometer data have significantly low amplitude on the order of $10^{-3}$. This makes it difficult to visualize in the following steps. As a result, the data have been scaled up by a factor of $10^{-3}$. In 2015, a study by KS Kumar et al. [65] showed that a digital FIR filter with Kaiser windowing performs better than other windowing techniques for noise removal in vital-sign electrical signals. With this consideration, Kaiser windowing was applied as an after-scaling step for noisy artifact removal from the raw dataset. The Kaiser window with parameter β=5.1 is taken to be constant throughout the study. The parameter β controls the immunity against spectral leakage. It is observed that if the β value is in the range from 5 to 10, it improves the signal immunity. However, higher β also dampens the amplitude, which is not preferable. As a result, an experimental trade-off between amplitude dampening and immunity against spectral leakage is considered, with β=5.1. Additionally, it should be noted that an extra step of using FIR or IIR filters in combination with windowing for more efficient noise removal was not considered in our work, since the data in the three considered positions—Resting, Apnea, and Valsalva—are already of quite good quality. Introducing additional filters would have increased the complexity of the algorithm with minimal performance gains. For the efficient study of interferometric signal properties, the signal was transformed from the time domain to the frequency domain using the Fast Fourier Transform (FFT). The FFT is computationally faster than the Discrete Fourier Transform (DFT). The one-sided power spectrum was consequently normalized and plotted as shown in Figure 3.

Often during breathing estimation by interferometers, the fundamental frequency peak turns out to be weaker than the second breathing harmonic peak, as is the case in the research reported by M. Mabrouk et al. [66] (the fundamental frequency and second breathing harmonic definition with localization are discussed in detail in Section 2.4). This is due to the presence of unwanted artifacts. To remove these, additional filters require to be used. In our study, consistently for all subject IDs, the fundamental frequency peak is higher than the second breathing harmonic peak. This is shown for one of the subjects in Figure 3. This validates that the signal pre-processing steps that were incorporated by the authors as described in Section 2.3 successfully removed the noisy artifacts.

### 2.4. Vital-Sign Signature Identification

The processed signal is fed into the randomizer block, as shown in Figure 2, dividing the dataset according to a 70:30 ratio to form the training and testing sets. With 30 participants, this corresponds to 21 training participants and 9 previously unseen testing participants. The split is selected to provide sufficient independent subjects for estimating the empirical feature distributions while retaining an independent test cohort for evaluating generalization. Each participant contributes measurements under the available physiological conditions, thereby preserving the scenario diversity within both subsets. Furthermore, the dataset is divided at the participant level rather than at the individual signal-window level to prevent measurements from the same participant from appearing simultaneously in training and testing subsets. This is particularly important for physiological radar sensing because multiple recordings from the same participant are correlated through subject-specific respiratory and cardiac characteristics.

The three available recordings, namely, Resting, Valsalva, and Apnea, are treated as distinct physiological recording conditions rather than as independent datasets. To systematically evaluate the human-identification capability, the recordings from the nine independent test participants are organized into reference–query comparisons. Specifically, the resting recording of each test participant is used as a reference observation, and this reference is compared with recordings from all nine test participants under the three available physiological conditions.

Thus, for each of the nine reference participants, there are 9×3=27 query observations. The total number of derived prediction instances is consequently as follows:

(7) $$\begin{array}{cc} N_{\mathrm{pred}} & =N_{\mathrm{ref}}\times N_{\mathrm{subject}}\times N_{\mathrm{scenario}}\\ \; & =9\times 9\times 3\\ \; & =243. \end{array}$$

The 243 prediction instances consist of same-subject and different-subject comparisons. For the same-subject class, each reference participant is compared with the three physiological recordings belonging to that same participant, giving

(8) $$\begin{array}{cc} N_{\mathrm{same}} & =N_{\mathrm{subject}}\times N_{\mathrm{scenario}}\\ \; & =9\times 3\\ \; & =27. \end{array}$$

For the different-subject comparisons, each reference participant is compared with the three recordings of each of the other eight participants, giving

(9) $$\begin{array}{cc} N_{\mathrm{different}} & =N_{\mathrm{subject}}\times \left(N_{\mathrm{subject}}-1\right)\times N_{\mathrm{scenario}}\\ \; & =9\times 8\times 3\\ \; & =216. \end{array}$$

Therefore,

(10) $$\begin{array}{cc} N_{\mathrm{pred}} & =N_{\mathrm{same}}+N_{\mathrm{different}}\\ \; & =27+216\\ \; & =243. \end{array}$$

The prediction instances are therefore generated through systematic reference–query enumeration rather than by generating synthetic physiological signals or artificially perturbing the measured features. The procedure does not increase the number of independent participants; rather, it exhaustively evaluates the valid reference–query comparisons available within the independent test cohort. Consequently, the 243 prediction instances should not be interpreted as 243 statistically independent subjects. The independent participant count remains nine for the testing cohort. For each reference–query pair, the physiological features extracted from the two recordings are compared using the proposed multi-feature decision framework. The ground-truth class is determined solely from the participant identity. Comparisons involving recordings from the same participant are assigned to the same-subject class, whereas comparisons involving different participants are assigned to the different-subject class. This procedure allows the physiological variability represented by the Resting, Valsalva, and Apnea conditions to be incorporated into the prediction analysis while maintaining strict participant-level separation between the training and testing data.

The final human-identification decision is based on a set of complementary physiological features extracted from the interferometric radar signal. The feature representation used for each reference–query comparison is defined as

(11) $$z={\left[\Delta f_{R},\;\Delta f_{H2},\;\Delta d_{H},\;D_{\mathrm{KL}}^{\mathrm{PS}},\;D_{J}^{\mathrm{HS}}\right]}^{T},$$

where $\Delta f_{R}$ denotes the absolute difference between the fundamental respiratory frequencies of the reference and query recordings, $\Delta f_{H2}$ denotes the absolute difference between their second respiratory harmonic frequencies, and $\Delta d_{H}$ denotes the absolute difference between their first inter-harmonic distances. The quantity $D_{\mathrm{KL}}^{\mathrm{PS}}$ represents the normalized Kullback–Leibler divergence between the normalized chest-displacement power spectra, while $D_{J}^{\mathrm{HS}}$ represents the Jensen-divergence-based feature obtained from the radar-derived cardiac signature.

The three frequency-domain respiratory features characterize the consistency of the fundamental respiratory rhythm and its harmonic structure. The chest-displacement spectral divergence provides a measure of similarity in the distribution of the spectral energy associated with thoracic motion. The cardiac feature provides complementary information from the heart-sound-related radar signature. Thus, the classifier does not operate directly on the raw radar waveform; instead, it operates on this physiologically interpretable feature vector.

For the respiratory features, the quantities supplied to the decision stage are calculated relative to the selected reference recording as

(12) $$\Delta f_{R}=\left|f_{R}^{\mathrm{query}}-f_{R}^{\mathrm{ref}}\right|,$$

(13) $$\Delta f_{H2}=\left|f_{H2}^{\mathrm{query}}-f_{H2}^{\mathrm{ref}}\right|,$$

and

(14) $$\Delta d_{H}=\left|d_{H}^{\mathrm{query}}-d_{H}^{\mathrm{ref}}\right|.$$

Accordingly, the feature vector contains both direct physiological frequency-discrepancy measures and distribution-based spectral similarity measures. Each feature is subsequently evaluated using the corresponding acceptance criterion described in the proposed multi-feature decision framework, and the resulting feature decisions are combined to determine whether the reference and query recordings originate from the same participant.

#### 2.4.1. Physiological Background and Uncertainty Modeling of Vital-Sign Signatures

The respiratory component of the measured chest displacement can be represented as a quasi-periodic physiological signal rather than as an ideal stationary sinusoid. A simplified representation is given by

(15) $$x_{R}\left(t\right)=A_{1}\left(t\right)\cos\left(2\pi f_{R}\left(t\right)t+\phi_{1}\right)+\sum_{k=2}^{K}A_{k}\left(t\right)\cos\left(2\pi kf_{R}\left(t\right)t+\phi_{k}\right),$$

where $f_{R}\left(t\right)$ denotes the instantaneous respiratory fundamental frequency, while $A_{k}\left(t\right)$ and $\phi_{k}$ denote the time-varying amplitude and phase of the *k*-th respiratory harmonic. The harmonic structure originates from the non-sinusoidal nature of thoracic displacement during inspiration and expiration. Consequently, the second respiratory harmonic is physiologically expected near $2f_{R}$, while the corresponding inter-harmonic spacing is expected near $f_{R}$.

For an ideal periodic respiratory displacement signal, the second harmonic and the first inter-harmonic distance satisfy

(16) $$f_{H2}=2f_{R},\;d_{H}=f_{H2}-f_{R}=f_{R},$$

where $f_{H2}$ denotes the second respiratory harmonic and $d_{H}$ denotes the first inter-harmonic distance. In measured interferometric radar signals, these equalities are not expected to hold exactly because respiration is quasi-periodic and affected by variations in respiratory effort, thoracic mechanics, posture, phase noise, finite observation duration, and spectral estimation uncertainty.

To explicitly quantify the deviation from the ideal harmonic relationship, the measured second-harmonic frequency is modeled as

(17) $${\hat{f}}_{H2}=2{\hat{f}}_{R}+\epsilon_{H},$$

where ${\hat{f}}_{R}$ and ${\hat{f}}_{H2}$ denote the measured fundamental and second-harmonic frequencies, respectively, and $\epsilon_{H}$ represents the combined physiological and measurement deviation from ideal harmonics. The corresponding measured inter-harmonic distance can therefore be expressed as

(18) $${\hat{d}}_{H}={\hat{f}}_{H2}-{\hat{f}}_{R}={\hat{f}}_{R}+\epsilon_{H},$$

The magnitude of the second-harmonic deviation is quantified by the residual

(19) $$e_{H2}={\hat{f}}_{H2}-2{\hat{f}}_{R},$$

while its normalized form is defined as

(20) $$e_{H2}^{\mathrm{rel}}=\frac{\left|{\hat{f}}_{H2}-2{\hat{f}}_{R}\right|}{2{\hat{f}}_{R}},$$

Under an ideal stationary periodic model, $e_{H2}=0$. Non-zero values quantify the deviation produced by physiological non-stationarity, non-sinusoidal respiratory morphology, spectral leakage, finite observation duration, and peak-estimation uncertainty. The normalized residual additionally allows the deviation to be compared across subjects with different respiratory rates.

Accordingly, the thresholds used in this work are not interpreted as universal physiological constants. Instead, they define an empirical uncertainty region around the theoretically expected relationships. The threshold interval is estimated from the inter-subject and intra-subject variability observed in the training cohort and subsequently fixed for evaluation of the independent test cohort. This approach preserves the physiological constraint imposed by the respiratory harmonic structure while accounting for the uncertainty inherent in non-contact radar measurements.

For the second harmonic, the search interval is therefore centered at the theoretically expected location $2f_{R}$. The experimentally observed tolerance accommodates deviations from ideal harmonic and spectral estimation uncertainty. Similarly, the threshold for fundamental-frequency similarity represents the observed variability around the reference respiratory frequency rather than assuming that respiratory frequency is invariant across physiological states.

#### 2.4.2. Fundamental Frequency

The fundamental frequency of breathing is extracted by identifying the most dominant peak in the entire power spectrum. This corresponds to the first breathing harmonic in the power spectrum of the chest displacement signal, highlighted by the red box in Figure 3. The fundamental frequency is recorded for each training subject across their three corresponding scenarios, as illustrated in Table 3.

**Figure 4:**
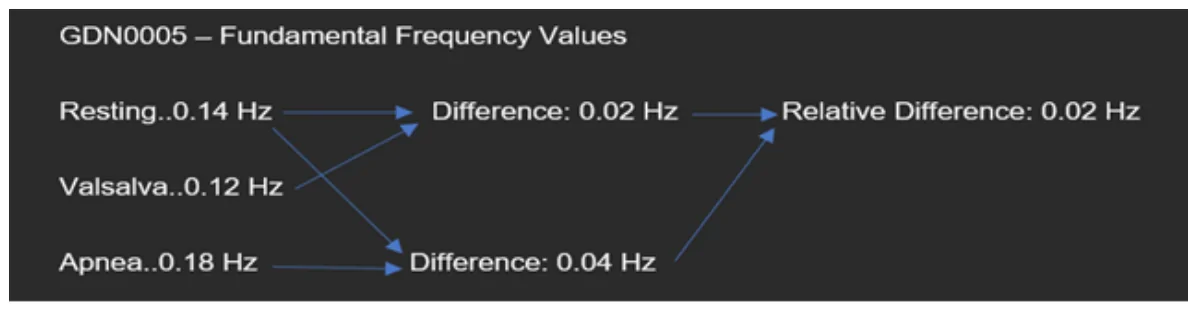
Relative difference between the fundamental respiratory frequencies of the reference and query recordings.

After this, the relative modulus difference in fundamental frequencies is obtained for each training subject until one single value is obtained, as shown below in Figure 4 for subject GDN0005.

#### 2.4.3. Second Breathing Harmonic

Let us assume that the fundamental frequency occurs at x=a Hz. The proposed algorithm then searches for the dominant peak in the range 2a±0.1 Hz, which corresponds to the second breathing harmonic. This is indicated by the green box in Figure 3. The second breathing harmonic is computed for each training subject in their respective three scenarios, as shown in Table 3. After this, the relative difference in the second breathing harmonic is obtained for each training subject until one single value is obtained (similar to Figure 4).

#### 2.4.4. First Inter-Harmonic Distance

The first inter-harmonic distance is defined as the difference (in Hz) between the fundamental frequency and the second breathing harmonic, depicted by the purple line in Figure 3. This inter-harmonic distance is computed for each training subject across their three corresponding scenarios, as illustrated in Table 3. After this, the relative difference in the first inter-harmonic distance is obtained for each training subject until one single value is obtained (similar to Figure 4).

#### 2.4.5. Chest Displacement Power Spectrum

The chest displacement power spectrum is shown in Figure 3. In this study, each power spectrum array is first converted into a probability distribution, followed by a padding mechanism to ensure all distributions have the same number of elements. The deviation between the frequency spectra of the same subjects is then quantified using the normalized Kullback–Leibler (KL) divergence, as shown in Equation (21). For each subject, the reference probability distribution is the one computed from the Resting position.

(21) $$D_{\mathrm{KL}}\left(P\;∥\;Q\right)=\frac{1}{N}\sum_{i=1}^{N}P\left(i\right)\;\log\frac{P(i)}{Q(i)},$$

where *P* represents the probability distribution of the current spectrum, *Q* is the reference distribution (computed from the Resting position), and *N* is the number of frequency bins.

The use of the KL divergence is motivated bythe need to treat the normalized power spectrum as a discrete energy distribution over frequency. Let $P(f_{i})$ and $Q(f_{i})$ denote two normalized power spectral distributions. The quantity $P(f_{i})$ represents the relative contribution of each frequency bin to the total chest-displacement spectral energy. KL divergence therefore measures the information-theoretic discrepancy between the spectral-energy distributions rather than comparing the absolute signal amplitude. This property is particularly relevant for interferometric chest-displacement signals because the absolute displacement amplitude may vary with the radar-to-subject distance, radar orientation, thoracic displacement magnitude, and individual physiology, whereas the distribution of spectral energy retains information about the structure of the cardio-respiratory motion. In this study, the resting spectrum is used as the subject-specific reference distribution, and the divergence quantifies how the spectral composition changes under the other physiological conditions. The interpretation is therefore not that KL divergence constitutes a physiological model by itself. Rather, it is used as a compact distributional descriptor of the spectral-shape deviation. Its suitability is consequently restricted to comparing non-negative, normalized power spectra represented on a common frequency grid.

The KL divergence score ranges from 0 to 1, where $D_{\mathrm{KL}}=0$ indicates identical distributions and $D_{\mathrm{KL}}=1$ indicates maximal deviation. The resulting KL divergence scores for each subject are summarized in Table 4.

#### 2.4.6. Heart Sound Extraction

**Figure 5:**
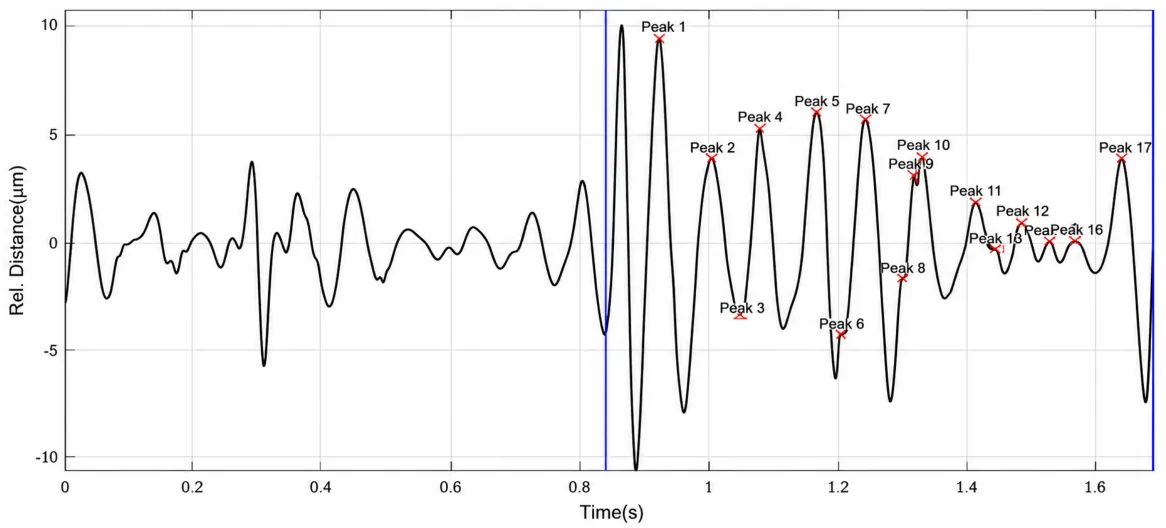
Heart sound extraction.

In this section, we study the heart sound as a potential vital-sign signature for use in human detection. The heart sound is extracted using the HSMM model [64] and its peaks are marked as shown in Figure 5).

The use of the hidden semi-Markov model (HSMM) is based on the temporal structure of cardiac acoustic events rather than on an assumption that the radar signal is acoustically equivalent to a phonocardiogram (PCG). Interferometric radar measures small periodic thoracic and cardiac-induced mechanical displacements, within which cardiac activity can produce temporally localized micro-motion components. The HSMM provides a state-based temporal representation in which individual cardiac events are characterized by both their observation likelihood and their state duration. The adaptation to radar interference signals is therefore based on temporal-event consistency. Radar-specific pre-processing is first applied to suppress measurement artifacts, after which, the HSMM is used to identify temporally structured cardiac events. The resulting heart-sound feature is consequently interpreted as a radar-observed cardiac mechanical signature rather than a direct acoustic heart-sound measurement. Because the present dataset does not provide an independent clinical reference for every radar-derived cardiac event, the HSMM extraction is treated as a feature-extraction component rather than as a clinically validated heart-sound diagnostic method. Its contribution to human detection is evaluated through the subsequent feature-consistency analysis and classification framework.

**Figure 6:**
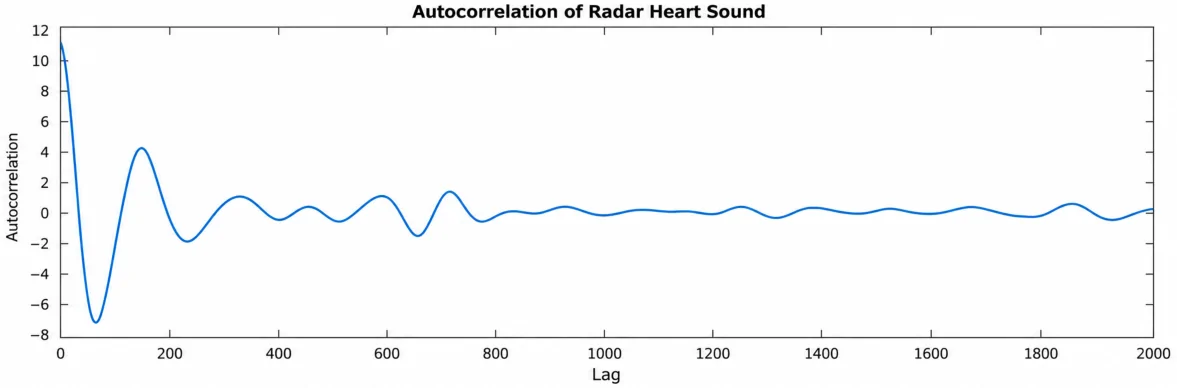
Autocorrelation function of extracted heart sound.

It has previously been suggested that human heart sounds can be used as a vital-sign signature [67]. Therefore, in this research an auto-correlation tool is incorporated to study the time axis similarity of the heart sound, as show in Figure 6.

The Jensen–Shannon divergence score is investigated between two auto-correlation functions of the same subject to find the deviation, as shown in Table 5.

#### 2.4.7. Multi-Feature Physiological Consistency and Contribution Model

The proposed framework combines complementary physiological features rather than relying on a single physiological signature. For a reference–query pair, the decision variables consist of the fundamental respiratory frequency deviation $\Delta f_{R}$, second respiratory harmonic deviation $\Delta f_{H2}$, inter-harmonic distance deviation $\Delta d_{H}$, normalized power-spectrum divergence $D_{\mathrm{KL}}^{\mathrm{PS}}$, and the normalized autocorrelation divergence $D_{J}^{\mathrm{HS}}$.

The multi-feature decision can therefore be expressed conceptually as

(22) $$D=F\left(\Delta f_{R},\;\Delta f_{H2},\;\Delta d_{H},\;D_{\mathrm{KL}}^{\mathrm{PS}},\;D_{J}^{\mathrm{HS}}\right),$$

where F(·) denotes the hierarchical physiological acceptance rules implemented in the proposed framework. A feature contributes to the final decision when its corresponding physiological consistency criterion is satisfied. Thus, the feature contribution is determined structurally through the decision hierarchy rather than through arbitrary equal weighting.

Importantly, the proposed framework does not assume that the contribution of each physiological feature can be represented by a universal scalar weight. Such weighting would require an additional statistical estimation procedure and sufficient independent data to reliably estimate feature-specific contributions. Given the limited number of independent participants in the present dataset, introducing additional fitted weights could result in unstable or dataset-dependent estimates. Instead, the feature contribution is defined structurally through the hierarchical decision function F(·), where each feature provides a distinct physiological constraint on reference–query consistency. This provides an interpretable theoretical model of feature contribution without introducing additional fitted parameters or compromising the subject-independent evaluation protocol.

The respiratory frequency and harmonic-related features constrain the consistency of respiratory dynamics, whereas the spectral and autocorrelation divergence features provide complementary distributional and temporal-structure information. The final decision therefore reflects the joint physiological consistency of multiple signatures rather than a single threshold. The thresholds used by the individual decision rules are determined during the development of the framework and remain fixed during evaluation. No test-set information is used to modify the decision criteria. This preserves the interpretability of the proposed rule-based framework while avoiding the introduction of additional fitted parameters that are not required by the implemented method.

## 3. Results

**Figure 7:**
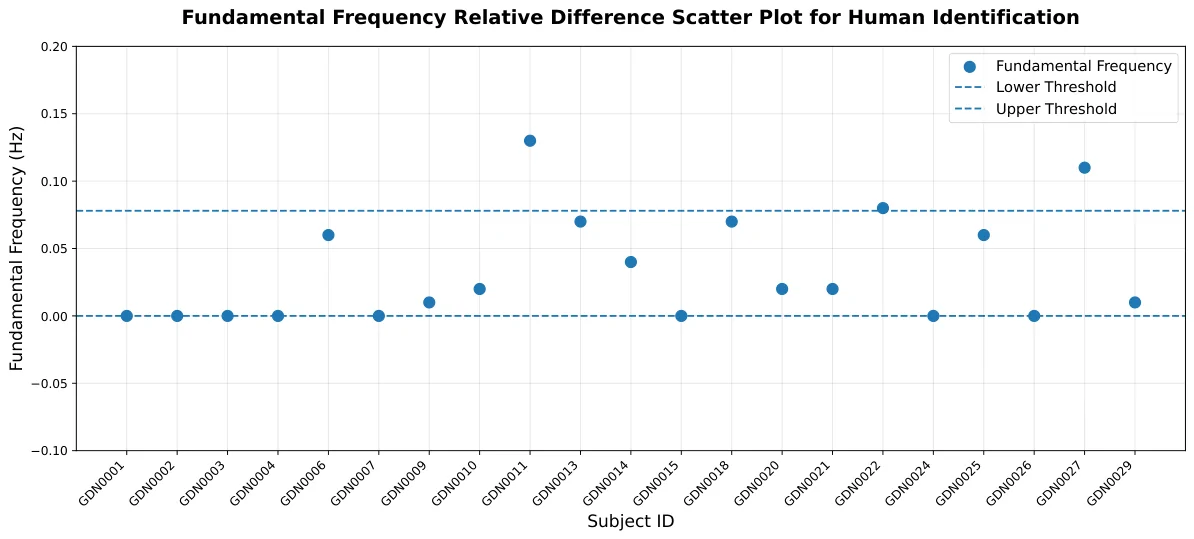
Fundamental frequency relative difference visualization.

**Figure 8:**
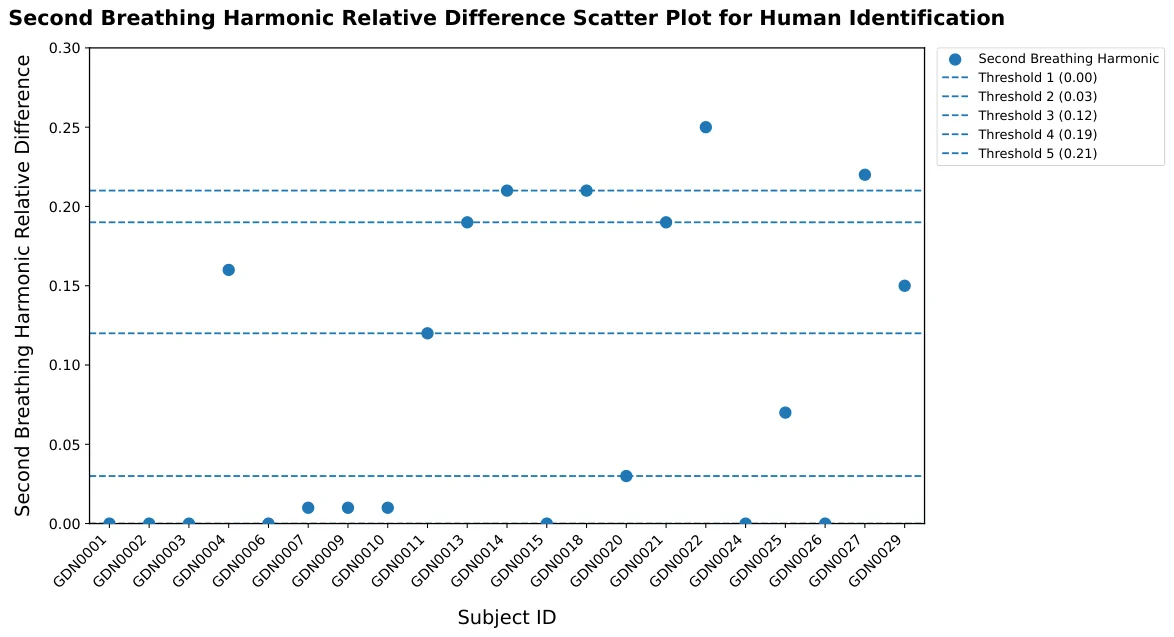
Second breathing harmonic relative difference visualization.

**Figure 9:**
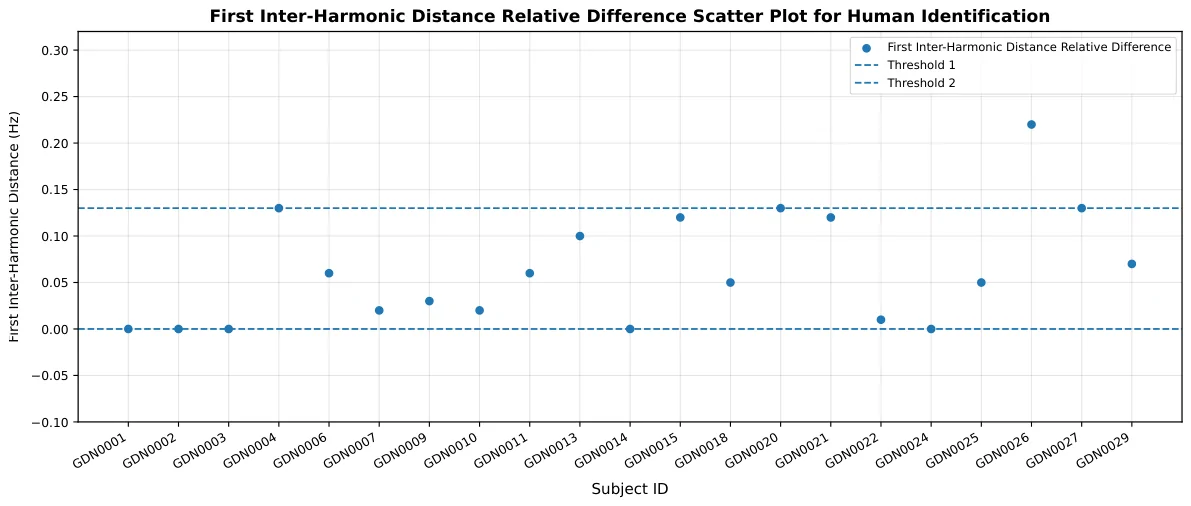
First inter-harmonic distance relative difference visualization.

The relative difference of the fundamental frequency is repeatedly computed as shown in Figure 4 on every training subject for their corresponding three scenarios. It is repeated similarly for the remaining vital-sign signatures: the second breathing harmonic and the first inter-harmonic distance. Lastly, the researchers performed a visualization of the final relative differences for the above three vital-sign signatures, as shown in Figure 7, Figure 8 and Figure 9.

As is qualitatively evident from Figure 7, the fundamental frequency relative difference for the same subjects predominantly falls along the black dotted threshold line of 0 Hz to 0.08 Hz. If Mr. A is a user, Mr. A’s unique fundamental frequency is extracted at a reference position. Then, the new fundamental frequency values of an unknown subject (could be Mr. A or someone else) are extracted. The relative difference algorithm is then run to find the relative differences of the two fundamental frequencies. If the relative difference value falls inside the observed threshold, then the unknown subject will be predicted to be Mr. A. Otherwise, it will be predicted to be another subject in the room. A similar approach is applied for the second breathing harmonic and the first inter-harmonic distance, as shown in Figure 8 and Figure 9.

**Figure 10:**
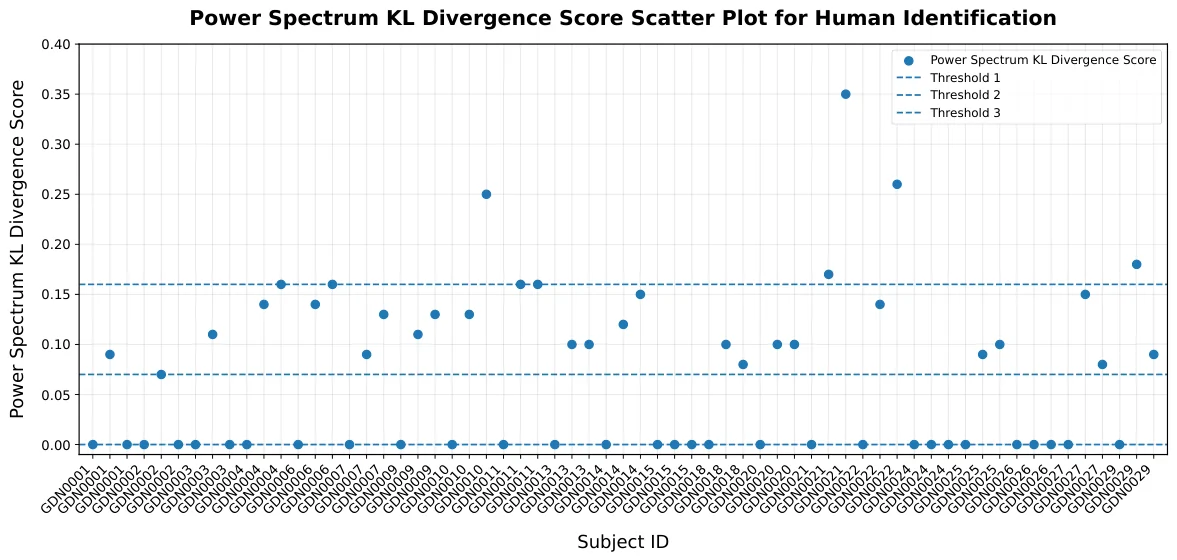
Normalized KL divergence score visualization of the power spectrum.

For the chest displacement spectrum, a qualitative thresholding is applied, as shown in Figure 10, with the human detection algorithm similarly to the above.

Up until now, multiple vital-sign signature thresholding has been identified which could prove effective for human detection. However, before this can be fed into ML prediction, one important question remains unanswered—that is, whether other physiological parameters of the subject concerned, such as age, height, weight, and BMI, also influence the vital-sign signatures. To find these answers, the statistical test results of the identified vital-sign signatures and their corresponding physiological parameters are considered. In this case, the dataset size for the vital-sign signatures is limited, which prevents reliable application of a two-sample *t*-test. Therefore, a non-parametric alternative with fewer distributional assumptions is adopted. Specifically, this study employs the Wilcoxon rank-sum test to evaluate whether the distributions of the vital-sign signatures and the corresponding physiological parameters differ significantly. This approach is suitable for small sample sizes and does not assume normality.

Table 6 below shows the investigation findings for the *p*-value and test statistic value of the Wilcoxon rank sum test between the vital-sign fingerprints and the physiological parameters—age, height, weight, and BMI.

From Table 6, it is evident that the *p*-values are considerably below the significance level of 0.05. Hence, the null hypothesis is rejected. Moreover, the positive test statistic score indicates that the first class has higher values compared to the later class.

From a physiological perspective, the extracted radar features are expected to depend primarily on instantaneous cardio-respiratory dynamics rather than directly on demographic variables. Age, height, weight, and BMI can influence respiratory mechanics and cardiovascular function indirectly; however, these variables do not impose a deterministic one-to-one mapping onto the instantaneous respiratory frequency, harmonic amplitude, or chest-displacement spectral structure. For example, the respiratory frequency is affected by autonomic regulation, respiratory effort, metabolic state, and behavioral control, while the measured chest displacement additionally depends on thoracic mechanics and radar-viewing geometry. Consequently, the absence of a simple monotonic relationship between a demographic parameter and an instantaneous radar signature would be physiologically plausible even when demographic characteristics influence the population distribution of respiratory behavior. The statistical analysis is therefore interpreted as an assessment of whether the extracted signatures exhibit systematic distributional dependence on the investigated demographic variables, rather than as evidence that demographic variables have no physiological influence whatsoever.

The identified vital-sign thresholds are fed into the prediction block along with the Wilcoxon rank sum test results and ground truths, as illustrated in Figure 2.

The prediction dataset comprises nine subjects, each with three distinct scenarios. Consequently, the total number of eligible combinations is 243. Of these, 218 were predicted correctly, resulting in an overall classification of 89.71% accuracy, 95.26% precision, 94.15% F1-score, and 93.06% sensitivity.

A direct quantitative comparison with previously reported radar-based physiological detection methods requires careful consideration because existing studies differ substantially in the sensing modality, radar configuration, participant population, physiological protocol, feature definition, and classification objective. Approaches reported in the literature [44,45,46,47,49,50,55] that address only respiration-rate or heart-rate estimation, or that employ substantially different sensing configurations and experimental objectives, are treated as related approaches rather than direct experimental baselines. The present work addresses a more specific human-identification problem based on the consistency of multiple physiological signatures extracted from interferometric radar measurements. Therefore, a numerical comparison of reported accuracy values across heterogeneous experimental conditions will not provide a methodologically equivalent assessment.

## 4. Discussion

The experimental results demonstrate that interferometric radar sensing can simultaneously perform non-contact human detection and health monitoring by exploiting multiple physiologically meaningful vital-sign signatures. Unlike conventional approaches that rely on a single respiratory or cardiac parameter, the proposed framework integrates complementary cardio-respiratory features derived through physiological modeling, thereby improving detection reliability under practical operating conditions. The incorporation of multiple physiological signatures rather than a single vital-sign parameter also constitutes an important design consideration. Human cardio-respiratory signals exhibit considerable variability across individuals and environmental conditions. Respiratory motion may become shallow, heart-related components may be partially masked by noise, and signal characteristics can vary with posture, stress level, or spontaneous body movement. Under such circumstances, reliance on a single physiological feature increases susceptibility to missed detections and false alarms. By integrating multiple complementary signatures, the proposed framework introduces measurement redundancy, allowing reliable detection even when individual features become weak or partially corrupted. Furthermore, simultaneously reproducing multiple harmonically consistent respiratory and cardiac signatures is substantially more difficult than mimicking a single physiological parameter, thereby providing an inherent degree of resistance against spoofing attacks. Multi-feature physiological fusion therefore improves both the robustness and security of the proposed non-contact human detection framework.

A noteworthy aspect of the proposed framework is the use of empirically optimized thresholds for physiological feature extraction. In theory, the respiratory fundamental frequency, higher-order breathing harmonics, and inter-harmonic distances exhibit deterministic mathematical relationships. However, physiological signals acquired under real-world conditions are inherently quasi-periodic, nonlinear, and time-varying. Variations in respiratory effort, body posture, subject-specific physiology, measurement noise, and the finite spectral resolution of the radar system introduce deviations from ideal harmonic behavior. Consequently, rigid theoretical thresholds may degrade detection performance by increasing false classifications. The experimentally optimized thresholds adopted in this study, under the supervision of healthcare professionals, therefore represent a practical compromise between physiological theory and measurement uncertainty, enabling improved robustness against inter-subject variability while maintaining reliable detection performance. These observations highlight the importance of physiologically realistic modeling when designing radar-based non-contact sensing systems.

Compared with conventional radar vital-sign sensing techniques, which predominantly estimate respiratory or heart rate from range-Doppler responses or dominant spectral peaks, interferometric sensing directly exploits phase coherence to measure sub-millimeter chest displacement. This capability enables reliable extraction of higher-order physiological characteristics, including displacement amplitude, harmonic content, and inter-harmonic relationships, rather than relying solely on frequency estimation. The resulting signature-level physiological representation provides richer discriminative information for human detection and remains effective under low-motion or unconscious conditions, where conventional Doppler-based approaches often exhibit degraded performance.

Moreover, an important advantage of the proposed methodology is that it preserves user privacy. Consequently, the system is inherently independent of ambient illumination, facial image quality, and many of the line-of-sight constraints that affect camera-based solutions. Furthermore, consolidating both human detection and physiological monitoring within a single interferometric radar platform reduces the dependence on heterogeneous sensing modalities commonly employed in smart-home environments. This simplification decreases system complexity, computational overhead, installation cost, and power consumption while improving the practicality of large-scale deployment.

While there are many positive aspects to the study, certain limitations are also recognized. The current study is limited to single-subject data acquisition conducted in a controlled clinical environment. This experimental design is intentionally selected to isolate interferometric chest displacement and cardio–respiratory harmonics, thereby enabling reliable identification of physiologically coherent vital-sign signatures without contamination from multi-path effects or inter-subject interference. While such controlled conditions facilitate foundational vital-sign signature modeling, they do not capture the full complexity of unconstrained multi-occupant smart systems. From a signal-processing perspective, simultaneous subjects introduce an additional differentiability problem because multiple quasi-periodic chest-displacement processes may occupy overlapping frequency bands. Thus, direct application of the present single-subject feature thresholds to an unresolved multi-person mixture is not assumed to be valid. The proposed framework should instead be regarded as the physiological-signature layer of a future multi-person system, where subject localization and separation precede signature extraction. This distinction is important because the present work establishes the physiological feature space under controlled conditions rather than claiming complete multi-person source separation. Last but not least, the relatively small cohort size is an important limitation of this study. The dataset contains 30 independent participants, with 21 used for training-based feature characterization and threshold determination and 9 previously unseen participants reserved for testing. Thus, the study provides an initial validation under controlled conditions but does not establish population-level generalization. Although the reference–query procedure produces 243 prediction instances, these do not represent 243 independent subjects; the reported performance is therefore interpreted with respect to the nine independent test participants.

Future work will extend the proposed interferometric sensing framework to multi-person scenarios through integration of spatial separation and localization techniques with a larger dataset. This will validate robustness under realistic residential conditions involving occlusion, subject motion, and environmental clutter. Additionally, a larger and more diverse dataset will be incorporated to further assess inter-subject variability and the long-term applicability of the identified vital-sign signatures from this work.

## 5. Conclusions

This study demonstrates a privacy-preserving interferometric radar framework that simultaneously performs human detection and physiological monitoring by modeling multi-feature vital-sign signatures. Clinical data from 30 healthy subjects are used to extract the fundamental frequency, second breathing harmonic, first inter-harmonic distance, chest displacement signal, and heart sounds. The extracted features were interpreted using physiologically determined relationships between the respiratory fundamental frequency, higher-order harmonics, inter-harmonic spacing, spectral-energy distribution, and cardiac mechanical activity. Rather than assuming ideal harmonics, the framework models deviations from the ideal relationships as a consequence of physiological non-stationary and measurement uncertainty. Practical decision regions were subsequently derived from the observed variability of the training cohort. Then, these features were fed into a machine learning module for validation. The prediction was performed on 243 test cases. The proposed pipeline achieved an overall human detection rate of 89.71% accuracy, 95.26% precision, 94.15% F1-score and 93.06% sensitivity. Consequently, the results establish inter-subject physiological variability but do not directly imply robustness to unresolved multi-person signal superposition.

The present study is intentionally restricted to single-subject acquisitions, although the dataset contains measurements from 30 independent participants. The study highlights several key outcomes: the interferometric approach allows simultaneous human detection and health monitoring; it is non-intrusive and independent of lighting, line-of-sight, and imaging resolution; it reduces the sensor count, computational complexity, and cost while improving energy efficiency; and it supports physiologically realistic multi-feature modeling to address the inherent variability in human signals. Since vital signs have been used as signatures, the model can detect unconscious human subjects in the case of search and rescue operations. Adaptive thresholds for the fundamental frequency, second breathing harmonic, and inter-harmonic spacing improve robustness against inter-subject variability, while multi-feature fusion compensates for weak or corrupted signals and will enhance anti-spoofing security in smart systems.

Overall, this work establishes a scalable, cost-efficient, and energy-aware interferometric sensing paradigm that fuses multiple vital-sign signatures for reliable human detection and physiological monitoring, providing a foundation for future work to extend this study to real-world smart-home use.

Future work will incorporate subject localization and source-separation mechanisms before physiological signature extraction. Hardware-induced phase errors will likewise be recognized as an uncertainty source and be mitigated through I/Q calibration and phase pre-processing.

## Figures and Tables

**Table 1:** Related work in the field of human detection.

Reference	Year	Modality	Privacy	Healthcare	Multi-Person	Environment
[2]	2021	Camera			✓	RW
[3]	2021	Camera			✓	RW
[4]	2021	Camera			✓	RW
[5]	2021	Camera			✓	RW
[6]	2022	Camera			✓	RW
[7]	2022	Camera			✓	RW
[8]	2022	Camera			✓	RW
[9]	2022	Camera			✓	RW
[10]	2023	Camera			✓	RW
[11]	2023	Camera			✓	RW
[12]	2023	Camera			✓	RW
[13]	2023	Camera			✓	RW
[14]	2023	Camera			✓	RW
[15]	2024	Camera			✓	RW
[16]	2024	Camera			✓	RW
[17]	2024	Camera			✓	RW
[18]	2024	Camera			✓	RW
[19]	2025	Camera			✓	RW
[20]	2025	Camera			✓	RW
[21]	2025	Camera			✓	RW
[22]	2025	Camera			✓	RW
[23]	2026	Camera			✓	RW
[24]	2022	Video			✓	RW
[25]	2021	RGBD			✓	RW
[26]	2021	Thermal	✓		✓	RW
[27]	2022	Thermal	✓		✓	RW
[28]	2022	Thermal	✓		✓	RW
[29]	2025	Thermal	✓		✓	RW
[30]	2025	IR	✓		✓	RW
[31]	2022	RGBD, T			✓	RW
[32]	2023	Camera+			✓	RW
[33]	2024	Camera, T			✓	RW

✓: Yes; IR: Infrared Camera, RW: Real-world, RGBD: Red-Green-Blue-Depth Camera, T: Thermal Camera.

**Table 2:** Related work in the field of human detection (contd.).

Reference	Year	Modality	Privacy	Healthcare	Multi-Person	Environment
[34]	2023	WiFi	✓		✓	RW
[35]	2023	WiFi	✓		✓	RW
[36]	2024	WiFi	✓		✓	RW
[37]	2024	WiFi	✓		✓	RW
[38]	2024	WiFi	✓		✓	RW
[39]	2021	RSSI	✓		✓	RW
[40]	2025	LTE	✓		✓	RW
[41]	2022	LiDAR	✓		✓	RW
[42]	2021	Radar	✓		✓	RW
[43]	2021	Radar	✓		✓	RW
[44]	2021	Radar	✓	✓	✓	RW
[45]	2021	Radar	✓	✓	✓	RW
[46]	2021	Radar	✓	✓	✓	RW
[47]	2021	Radar	✓	✓	✓	RW
[48]	2022	Radar	✓		✓	RW
[49]	2022	Radar	✓	✓	✓	RW
[50]	2022	Radar	✓	✓	✓	RW
[51]	2023	Radar	✓		✓	RW
[52]	2024	Radar	✓		✓	RW
[53]	2024	Radar	✓		✓	RW
[54]	2024	Radar	✓		✓	RW
[55]	2024	Radar	✓	✓	✓	RW
[56]	2025	Radar	✓		✓	RW
[57]	2025	Radar	✓		✓	RW
[58]	2025	Radar	✓		✓	RW
[59]	2025	Radar	✓		✓	RW
[60]	2022	RF	✓		✓	RW
[61]	2022	RF	✓		✓	RW
[62]	2023	RF	✓		✓	RW
**Ours**	2026	I	✓	✓		C

✓: Yes; C: Clinical, I: Interferometric Sensing, LTE: Long-Term Evolution, RF: Radio-frequency, RSSI: Received Signal Strength Indicator, RW: Real-world.

**Table 3:** Fundamental frequency, second breathing harmonic, and first inter-harmonic distance for all training subjects.

Subject ID	Scenario	Fundamental Frequency (Hz)	Second Breathing Harmonic (Hz)	First Inter-Harmonic Distance (Hz)
GDN0001	Resting	0.1202	0.2140	0.0937
GDN0001	Valsalva	0.0505	0.2259	0.1753
GDN0001	Apnea	NA	NA	NA
GDN0002	Resting	0.1214	0.2233	0.1019
GDN0002	Valsalva	0.2506	0.5053	0.2547
GDN0002	Apnea	NA	NA	NA
GDN0003	Resting	0.0952	0.2178	0.1227
GDN0003	Valsalva	0.0987	0.2015	0.1028
GDN0003	Apnea	NA	NA	NA
GDN0004	Resting	0.1660	0.4427	0.2768
GDN0004	Valsalva	0.1066	0.2371	0.1360
GDN0004	Apnea	0.2145	0.4969	0.2825
GDN0006	Resting	0.1997	0.4109	0.2111
GDN0006	Valsalva	0.0889	0.2024	0.1134
GDN0006	Apnea	0.1452	0.2018	0.0566
GDN0007	Resting	0.1071	0.2457	0.1386
GDN0007	Valsalva	0.1062	0.2158	0.1097
GDN0007	Apnea	0.1020	0.2899	0.1879
GDN0009	Resting	0.1276	0.2063	0.0787
GDN0009	Valsalva	0.1319	0.2027	0.0708
GDN0009	Apnea	0.1055	0.2111	0.1056
GDN0010	Resting	0.2137	0.4631	0.2495
GDN0010	Valsalva	0.1267	0.2178	0.0911
GDN0010	Apnea	0.1472	0.2266	0.0793
GDN0011	Resting	0.3036	0.6025	0.2989
GDN0011	Valsalva	0.1516	0.4416	0.2900
GDN0011	Apnea	0.2831	0.6492	0.3661
GDN0013	Resting	0.2148	0.4258	0.2110
GDN0013	Valsalva	0.1248	0.2298	0.1050
GDN0013	Apnea	0.1929	0.4132	0.2204
GDN0014	Resting	0.2104	0.4573	0.2469
GDN0014	Valsalva	0.1788	0.4210	0.2422
GDN0014	Apnea	0.1350	0.2106	0.0755
GDN0015	Resting	0.1123	0.2066	0.0943
GDN0015	Valsalva	NA	NA	NA
GDN0015	Apnea	NA	NA	NA
GDN0018	Resting	0.1924	0.4230	0.2306
GDN0018	Valsalva	0.2016	0.4124	0.2108
GDN0018	Apnea	0.1153	0.2096	0.0943
GDN0020	Resting	0.1851	0.4062	0.2211
GDN0020	Valsalva	0.1609	0.4448	0.2839
GDN0020	Apnea	0.1864	0.4174	0.2310
GDN0021	Resting	0.2951	0.6222	0.3271
GDN0021	Valsalva	0.2531	0.6020	0.3488
GDN0021	Apnea	0.2379	0.4110	0.1731
GDN0022	Resting	0.2272	0.4517	0.2245
GDN0022	Valsalva	0.2000	0.4521	0.2521
GDN0022	Apnea	0.1289	0.2082	0.0793
GDN0024	Resting	0.2457	0.4243	0.1785
GDN0024	Valsalva	NA	NA	NA
GDN0024	Apnea	NA	NA	NA
GDN0025	Resting	0.0692	0.2772	0.2080
GDN0025	Valsalva	0.0904	0.2003	0.1100
GDN0025	Apnea	0.1569	0.4156	0.2587
GDN0026	Resting	0.1815	0.4080	0.2265
GDN0026	Valsalva	NA	NA	NA
GDN0026	Apnea	NA	NA	NA
GDN0027	Resting	0.1820	0.4444	0.2623
GDN0027	Valsalva	0.0783	0.2140	0.1357
GDN0027	Apnea	0.1849	0.4533	0.2684
GDN0029	Resting	0.1611	0.3085	0.1474
GDN0029	Valsalva	0.1517	0.4696	0.3179
GDN0029	Apnea	0.1687	0.4713	0.3026

NA: Data not available.

**Table 4:** Normalized KL divergence of the chest displacement power spectrum.

Subject ID	Resting	Valsalva	Apnea
GDN0001	0.00	0.09	NA
GDN0002	0.00	0.07	NA
GDN0003	0.00	0.11	NA
GDN0004	0.00	0.14	0.16
GDN0006	0.00	0.14	0.16
GDN0007	0.00	0.09	0.13
GDN0009	0.00	0.11	0.13
GDN0010	0.00	0.13	0.25
GDN0011	0.00	0.16	0.16
GDN0013	0.00	0.10	0.10
GDN0014	0.00	0.12	0.15
GDN0015	0.00	NA	NA
GDN0018	0.00	0.10	0.08
GDN0020	0.00	0.10	0.10
GDN0021	0.00	0.17	0.35
GDN0022	0.00	0.14	0.26
GDN0024	0.00	NA	NA
GDN0025	0.00	0.09	0.10
GDN0026	0.00	NA	NA
GDN0027	0.00	0.15	0.08
GDN0029	0.00	0.18	0.09

NA: Data not available.

**Table 5:** Jensen–Shannon divergence of the autocorrelation function of heart sound.

Subject ID	Resting	Valsalva	Apnea
GDN0001	0.00	−0.25	NA
GDN0002	0.00	0.27	NA
GDN0003	0.00	−0.11	NA
GDN0004	0.00	−0.16	−0.30
GDN0006	0.00	−0.01	−0.02
GDN0007	0.00	0.42	−0.02
GDN0009	0.00	0.02	−0.01
GDN0010	0.00	−0.11	−0.04
GDN0011	0.00	0.52	7.60
GDN0013	0.00	3.66	−4.41
GDN0014	0.00	0.02	0.03
GDN0015	0.00	NA	NA
GDN0018	0.00	−0.58	−0.46
GDN0020	0.00	−0.09	0.22
GDN0021	0.00	−0.01	0.02
GDN0022	0.00	0.06	0.15
GDN0024	0.00	NA	NA
GDN0025	0.00	12.71	2.79
GDN0026	0.00	NA	NA
GDN0027	0.00	2.76	−1.32
GDN0029	0.00	0.74	0.38

NA: Data not available.

**Table 6:** Wilcoxon rank-sum test results between the vital-sign features and the physiological parameters.

Feature Pair	*p*-Value	Test Statistic
Fundamental Frequency, Age	$2.0118\times 10^{-28}$	4284
Fundamental Frequency, Height	$1.805\times 10^{-20}$	11,385
Fundamental Frequency, Weight	$4.7265\times 10^{-31}$	4095
Fundamental Frequency, BMI	$6.4868\times 10^{-25}$	4542
Second Breathing Harmonic, Age	$4.1662\times 10^{-31}$	4095
Second Breathing Harmonic, Height	$1.805\times 10^{-20}$	11,385
Second Breathing Harmonic, Weight	$4.7265\times 10^{-31}$	4095
Second Breathing Harmonic, BMI	$4.7981\times 10^{-31}$	4095
First Inter-Harmonic Distance, Age	$1.271\times 10^{-23}$	4647
First Inter-Harmonic Distance, Height	$1.805\times 10^{-20}$	11,385
First Inter-Harmonic Distance, Weight	$4.7265\times 10^{-31}$	4095
First Inter-Harmonic Distance, BMI	$6.2318\times 10^{-16}$	5319

## Data Availability

The dataset used in this study has already been cited.
